# Treating Arterial Ageing in Patients with Diabetes: From Mechanisms to Effective Drugs

**DOI:** 10.3390/ijms22062796

**Published:** 2021-03-10

**Authors:** Mojca Lunder, Miodrag Janić, Mišo Šabovič

**Affiliations:** 1 Department of Endocrinology, Diabetes and Metabolic Diseases, University Medical Centre Ljubljana, Zaloška cesta 7, SI-1000 Ljubljana, Slovenia; mojca.lunder@kclj.si (M.L.); miodrag.janic@kclj.si (M.J.); 2 Faculty of Medicine, University of Ljubljana, Vrazov trg 2, SI-1000 Ljubljana, Slovenia; 3 Department of Vascular Diseases, University Medical Centre Ljubljana, Zaloška cesta 7, SI-1000 Ljubljana, Slovenia

**Keywords:** diabetes, arterial ageing, endothelial dysfunction, arterial stiffness, expression of longevity genes, antidiabetic drugs, antiaging approach

## Abstract

Diabetes mellitus is a major healthcare problem. It is not only characterized by hyperglycemia and chronic complications, but in longer lasting diabetes and a longer living population, it is also associated with accelerated arterial ageing, which importantly contributes to cardiovascular complications. The accelerated arterial ageing in patients with diabetes should be considered separately from arterial ageing in patients without diabetes. Basic and clinical research have allowed better insight into the mechanisms of arterial ageing. In a simplified mechanistic way, it could be considered that the three tightly connected cornerstone characteristics of arterial ageing in patients with diabetes are: phenotypic presentation as endothelial dysfunction and arterial stiffness, and the underlying basic ageing-facilitating mechanism represented as the impaired expression of genetic longevity pathways. Currently, specific drugs for preventing/treating arterial ageing are not available. Therefore, we aimed to review the capacity of available drugs, particularly antidiabetic drugs, to interfere with the arterial ageing process. In the near future, these characteristics could help to guide therapy in patients with diabetes. Overall, it appears that arterial ageing could become a new target in diabetes. The expanding knowledge regarding the capability of antidiabetic drugs and other available drugs to inhibit/delay arterial aging is therefore essential.

## 1. Introduction

The prevalence of diabetes mellitus is increasing globally. Due to sustained hyperglycemia and its chronic complications, diabetes represents one of the major healthcare problems worldwide [1,2]. Microvascular complications, such as retinopathy, nephropathy and neuropathy, significantly impair quality of life [1,2]. On the other hand, the majority of patients with diabetes die from macrovascular complications, such as myocardial infarction, heart failure, stroke, etc., that are mainly due to atherosclerotic cardiovascular disease. Cardiovascular diseases appear 10–15 years earlier in patients with diabetes compared to people without diabetes [1]. Diabetes increases the risk of death from cardiovascular diseases 2-6-fold [2]. Prevalence of diabetes increases with age; it is present in over 25% of adults older than 65 years. Furthermore, 50% of people with diabetes are older than 65 years [3]. Despite the plethora of therapies used to combat diabetes and its complications in primary and secondary prevention, the cardiovascular morbidity and mortality in these patients remains unacceptably high [4]. Thus, new targets for cardiovascular prevention in patients with diabetes should be identified. The most obvious of these is arterial ageing. It is well-known that arterial ageing is accelerated in diabetes and that it has detrimental cardiovascular effects [5]. These harmful effects are due to the increased susceptibility of aged arterial walls to the development and progression of atherothrombotic processes, as well as impaired hemodynamics characterized by the elevated central blood pressure, increased pulse pressure, and increased pulsatility that collectively lead to increased susceptibility to atherosclerosis and target organ damage [6].

Therefore, in addition to glycemic control, lipids, and hypertension, the arterial system, particularly its impairment due to accelerated ageing, could be focused upon in order to decrease the level of cardiovascular morbidity and mortality in diabetes [4].

Several antidiabetics and other drugs have been shown to improve arterial function and have the potential to prevent/delay arterial ageing [7]. Both, the process of arterial aging in patients with diabetes and potentially beneficial drugs are reviewed in the present manuscript. Our aim was to describe the potential of available drugs, since they could already be used in the treatment of diabetes. Taking into account arterial ageing, these data could shed some new light on the prevention of cardiovascular morbidity and mortality in patients with diabetes.

## 2. Arterial Ageing

At the microscopic level, there are two main components of arterial ageing that perpetuate one another and lead to pathological positive feedback. These are oxidative stress and low-grade inflammation [8,9,10]. The most important deleterious effect of oxidative stress is inactivation of endothelium nitric oxide synthase (eNOS), which is the key regulatory enzyme responsible for nitric oxide (NO) production. NO is the most important vasodilator in the endothelium and its abundant availability is synonymous with healthy endothelium. Thus, its impaired bioavailability leads to age-related reduction in endothelium-dependent vasodilation, enhanced vasoconstriction, and dysregulation of tissue perfusion [8,11,12]. Low-grade inflammation is another hallmark of arterial aging. It is generated by the pro-inflammatory milieu of endothelial cells (EC) and vascular smooth muscle cells (VSMC) [13]. This pro-inflammatory milieu consists of inflammatory cytokines, chemokines, adhesion molecules, and inducible NOS (iNOS) that orchestrate the microenvironment. The main molecule connecting the processes of oxidative stress and low-grade inflammation is the nuclear factor κB (NFκB) [14,15].

In addition, accumulation of dysfunctional mitochondria, due to their decreased turnover, contributes significantly to arterial ageing and the above-described microscopic ageing processes. This accumulation is characterized by means of mitochondrial dysfunction, i.e., diminished mitochondrial biogenesis, mitochondrial DNA damage, and respiratory chain dysfunction [16,17]. NFκB in ECs and VSMCs is again the main junctional point connecting mitochondria-derived oxidative stress with low-grade inflammation [18].

Moreover, the ability to combat molecular stresses and return to homeostasis is diminished in aged vascular cells. This is due to significant inactivation of Nrf2-driven antioxidant defense pathways, meaning that cells are constantly bathed in a pro-oxidative and pro-inflammatory microenvironment [18,19]. Aging also affects evolutionarily highly conserved cellular energy sensing pathways that regulate fundamental ageing processes by controlling cellular responses to nutrient availability and growth signals. These include the mechanistic/mammalian target of rapamycin (mTOR), AMP-activated protein kinase (AMPK), Klotho and sirtuins pathways. The enhancement of the mTOR pathway that is characteristic of ageing leads to increased endothelial cell senescence and endothelial dysfunction. On the other hand, sirtuins and AMPK pathways are inhibited, also leading to endothelial dysfunction, reduced NO bioavailability, and inflammation [20,21,22,23]. Overall, the impairment of these genetic longevity pathways is the essential mechanism underlying arterial ageing.

Cellular senescence is also one of the fundamental processes and consequences in arterial ageing. Endothelial cell senescence leads to endothelial dysfunction. Senescent cells change their function, thus contributing to a local pro-inflammatory milieu and microenvironmental changes. Additionally, their replicative abilities are altered, leading to diminished regeneration and angiogenic potential of the endothelium [24,25]. On the other hand, VSMC senescence is a crucial process in arterial stiffness. Senescent VSMCs change from flexible to a more rigid state, with changes in their secretome leading to deposition of less elastin and more crosslinked collagen [10].

Additionally, all of the above-described pathological ageing characteristics converge as ageing of the extracellular matrix that acts as a structural and regulatory element of the arterial wall. Its impairment leads to reduced elasticity and resilience, and to mechanical damage, in addition to reduced vascular mechanotransduction allowing for inappropriate hemodynamic alterations, pathological remodeling, and disruption of structural integrity [10].

At the macroscopic level, arterial ageing is characterized by changes in arterial structure and function, the most typical being intima and media thickening, increase in arterial stiffness, dilatation of the arterial lumen, and endothelial dysfunction [26]. Aged arteries lose their ability to respond adequately to various stimuli, such as blood pressure variations and cushioning of these variations when translated to small vasculature. Consequently, microvascular changes and rarefication occur, leading to various forms of tissue damage [10].

## 3. Interplay between Diabetes Mellitus and Arterial Ageing

Diabetes mellitus is characterized by chronic hyperglycemia and in type 2 diabetes by insulin resistance, i.e., by chronic hyperinsulinemia [27]. Endothelial dysfunction represents one of the earliest vascular complications in patients with diabetes. The endothelial dysfunction in diabetes deteriorates more rapidly in patients with type 2 diabetes compared to type 1, probably due to the toxic effect of hyperinsulinemia on the vascular wall. Endothelial dysfunction gradually leads to increased arterial stiffness [4,27]. It should be emphasized that both endothelial function as well as arterial elasticity deteriorate with age, the effect being multiplied when diabetes acts concomitantly.

The bidirectional association of oxidative stress and low-grade inflammation is also in the midst of responsible mechanisms for the deterioration of arterial structure and function in ageing diabetes arteries. NFκB represents the main molecule, which is also indirectly influenced by chronic hyperglycemia through reactive oxygen species (ROS) [6,28]. This particular molecule allows for the positive feedback association between diabetes and ageing. Additional damage is caused by diabetes in ageing arteries through downregulated anti-inflammatory and antioxidant pathways [3,6]. Basically, it could be assumed that inflammation and oxidative stress are differently activated in diabetes compared to arterial ageing, i.e., insulin resistance activating them in diabetes and impaired genetic longevity pathway in arterial ageing.

**Figure 1:**
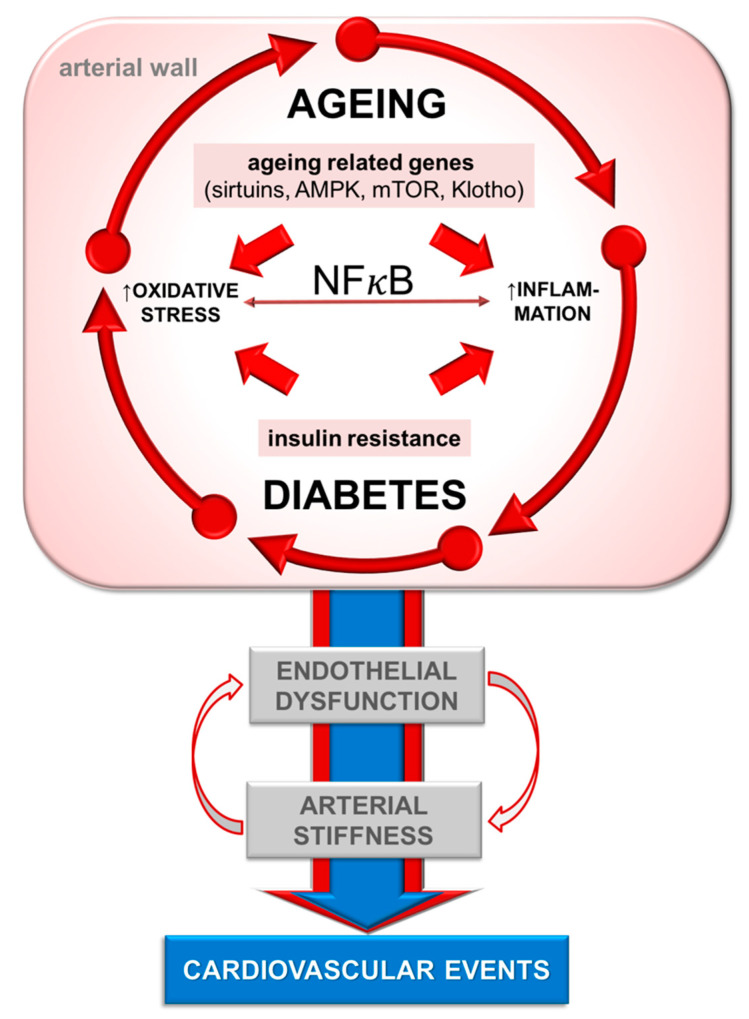
Interplay of mechanisms of arterial ageing in diabetes. NFκB—nuclear factor κB; AMPK—AMP-activated protein kinase; mTOR—mechanistic/mammalian target of rapamycin.

Therefore, diabetes influences all of the above-described mechanisms of aging (Figure 1). It has been hypothesized that the effect of diabetes and ageing itself (a) share the same mechanisms and thus lead to additional damage of the arterial wall; or (b) that they act together to amplify the deterioration of arterial function and structure caused by ageing, in addition to its own deleterious mechanisms, which directly influence the arterial wall. Based on the available data, the second hypothesis seems to be more correct [6]. Thus, it can be deduced that in middle-aged or older patients, arterial ageing per se acts as the primary failure of the arterial wall, and diabetes acts as the secondary arterial wall failure, seeded in the ageing milieu. Or in other words, primary cellular dysfunction is caused by ageing and senescence, amplified by secondary damage caused by hyperglycemia and insulin resistance in diabetes, the so-called secondary cell dysfunction. In younger patients, the situation is opposite. Primary cellular dysfunction is caused by diabetes and secondary by ageing. Although it may sound arbitrary, these two scenarios most likely significantly overlap. Nevertheless, these assumptions increase the probability of arterial wall dysfunction due to ageing in patients with diabetes.

Taking the complex interplay between diabetes and ageing into account, there might be some grounds for the beneficial effect of targeting arterial ageing as a different antidiabetic treatment for the prevention of deterioration of arterial wall structure and function in patients with diabetes.

## 4. Deleterious Effects of Arterial Ageing

**Figure 2:**
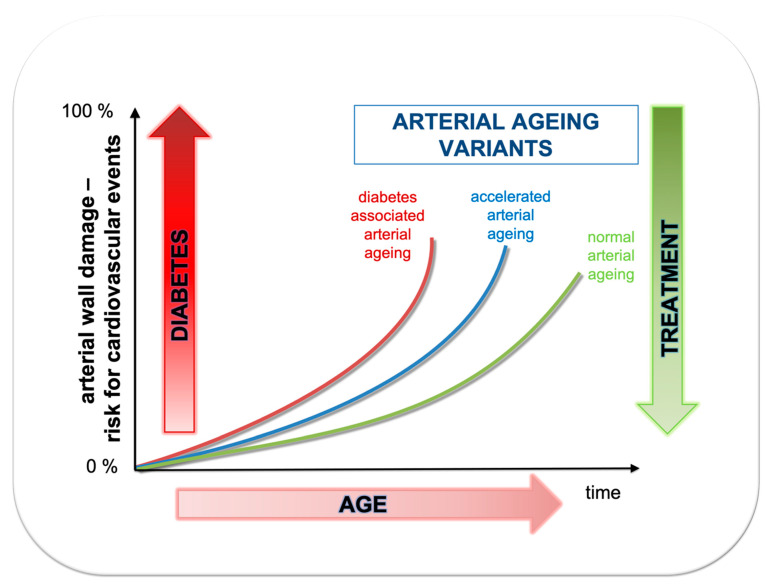
Association between age (time) and risk for cardiovascular events depending whether arterial ageing is normal, accelerated or additionally accelerated due to diabetes.

Ageing induces several complex structural and functional arterial wall impairments that are different, at least according to etiology and underlying mechanisms, compared to the direct effects of diabetes. Aged endothelium and age-related endothelial dysfunction lead to aggravation of the atherosclerotic process in the arterial wall. In addition, pro-thrombotic and pro-inflammatory pathways are overactivated. These changes collectively lead to an increased rate of atherothrombotic events (Figure 2) [29]. In addition to increased susceptibility to atherothrombosis, arterial ageing leads to increased arterial stiffness due to the deleterious impairment of hemodynamics. Consequently, central systolic pressure is increased, and central diastolic pressure decreased. Increased systolic pressure leads to increased afterload and due to ventricular-aortic coupling to left ventricular hypertrophy and diastolic dysfunction. Importantly, diastolic dysfunction of the left ventricle is a characteristic finding in patients with diabetes. Furthermore, decreased central diastolic pressure results in reduced coronary perfusion. Increased left ventricle demand and decreased coronary perfusion could result in coronary ischemia and coronary events. Furthermore, arterial ageing, via increased arterial stiffness leads to increased pulse pressure and increased pulsatility, which impair microcirculation in high-volume perfusion organs such as the kidneys and brain, thereby producing dysfunctions which lead to renal failure and cerebral white-matter lesions [30]. Thus, due to the interplay and overlap between ageing and diabetes, arterial ageing and its consequences are significantly aggravated in patients with diabetes.

## 5. Effect of Different Drugs on Arterial Ageing in Diabetes

### 5.1. Antidiabetic Drugs

#### 5.1.1. Metformin

Metformin is still recommended as the first-line pharmacological therapy in type 2 diabetes patients [31,32]. The main mechanism of its action is inhibition of the mitochondrial respiratory chain, thus increasing the AMP/ATP ratio and activating AMPK [31,33]. Metformin could also activate AMPK through an ATP-independent pathway or via inhibition of mTOR and the blockage of vacuolar lysosome ATP-ase [33]. Metformin inhibits gluconeogenesis and fatty acid synthesis in the liver and stimulates glucose uptake by translocation of glucose transporter 4 to the cell membrane in skeletal muscle [34].

In several studies, metformin was shown to improve endothelial function in type 2 diabetes patients [35,36,37]. It also decreased the concentration of vascular cell adhesion molecule-1 (sVCAM-1) and increased asymmetric dimethylarginine in patients with type 2 diabetes and stable coronary artery disease [36]. In another study, metformin was shown to significantly reduce the levels of sVCAM-1, von Willebrand factor (vWF), tissue-type plasminogen activator (t-PA), plasminogen activator inhibitor-1 (PAI-1) and soluble intercellular adhesion molecule-1 (sICAM-1) in patients with type 2 diabetes mellitus. The above listed laboratory parameters are associated with endothelial function [38]. However, other studies failed to show improvement of endothelial function after metformin treatment [39,40].

Only a few studies explored the effects of metformin on arterial stiffness and showed a potentially beneficial effect in type 2 diabetes patients [41,42]. Additionally, metformin was shown to decrease arterial stiffness in patients with non-alcoholic fatty liver disease (NAFLD) or young women with polycystic ovary syndrome (PCOS) [43,44]. However, in other studies, there was no metformin effect on arterial stiffness parameters [45,46].

Metformin increased sirtuin 1 protein expression and mTOR expression in peripheral blood mononuclear cells from subjects with prediabetes [47]; sirtuin 1 expression was also induced in patients with carotid artery atherosclerosis [48]. One of the basic mechanisms of action of metformin is the inhibition of mTOR [33]. Metformin was shown to act as an anti-inflammatory by suppression of NFκB via AMPK dependent pathways. Additionally, it decreased the formation of ROS through mTOR, leading to a reduction of superoxide that could cause DNA damage [35].

In the UK Diabetes Perspective Study (UKPDS), metformin decreased the risk for myocardial infarction by up to 16% [49]. It was shown to decrease cardiovascular mortality, all-cause mortality, and incidence of cardiovascular events in patients with coronary artery disease and type 2 diabetes mellitus [50]. Another meta-analysis showed that metformin acted favorably on all-cause mortality, cardiovascular death, and myocardial infarction, the differences not being statistically significant [51].

#### 5.1.2. SGLT-2 Inhibitors

Sodium-glucose cotransporter-2 (SGLT-2) inhibitors promote urinary glucose excretion and decrease plasma glucose concentration through the inhibition of SGLT-2 channels in the proximal renal tubules. This leads to inhibition of glucose reabsorption [52]. SGLT-2 inhibitors improve glycemic control through decrease of glycated hemoglobin levels (HbA1c) by up to 0.7–1.0%; the effect is insulin-independent. Additionally, a decrease of body weight by approximately 2–3 kg due to induction of negative caloric balance is observed [52].

Dapagliflozin and empagliflozin improved endothelial dysfunction in various studies [46,53,54]. In another study dapagliflozin improved endothelial dysfunction through increase of brachial artery flow-mediated dilation and reactive hyperemia index [52]. The beneficial effects of SGLT-2 inhibitors on endothelial function were proven in the majority of studies, though not all of them [55].

Treatment with SGLT-2 inhibitors reduced systolic blood pressure by 4–6 mmHg and diastolic blood pressure by 1–2 mmHg. Improvement of arterial stiffness with SGLT-2 inhibitors could be attributed to relaxation of vascular smooth muscle [56]. SGLT-2 inhibitors were shown to improve parameters of arterial stiffness, such as reduction of central systolic blood pressure, central pulse pressure [57,58], pulse wave velocity [46,52] and augmentation index [59] in patients with diabetes.

The effects of SGLT-2 inhibitors on the expression of longevity genes have not yet been studied in detail. According to the publication of Packer, one of the possible mechanisms of SGLT-2 inhibitors could be activation of sirtuin 1 and AMPK signaling pathways and suppression of the Akt/mTOR pathway [60]. However, additional studies are needed to prove these very reliable, but still hypothetical effects.

Several studies of cardiovascular outcome effects have proven the beneficial effects of SGLT-2 inhibitors [32,61,62]. EMPA-REG OUTCOME (Empagliflozin Cardiovascular Outcome Event Trial in Type 2 Diabetes Mellitus patients) was the first major study, proving that empagliflozin significantly decreases the primary composite outcome—3-point MACE (composed of cardiovascular death, nonfatal myocardial infarction and non-fatal stroke) in patients with diabetes, by 14% [63]. Similarly, canagliflozin in the CANVAS study (Canagliflozin and Cardiovascular and Renal Events in Type 2 Diabetes) decreased the incidence of 3-point MACE by 14% [61]. However, dapagliflozin in DECLARE-TIMI (Dapagliflozin and Cardiovascular Outcomes in Type 2 Diabetes) decreased the incidence of 3-point MACE by 7% in patients with diabetes, but the effect was not significantly different compared to placebo. On the other hand, the effect of dapagliflozin on cardiovascular death and hospitalization due to heart failure was superior compared to placebo [62,64].

#### 5.1.3. GLP-1 Receptor Agonists

GLP-1 (glucagon like peptide-1) agonists act through the incretin system. In the latter, GLP-1 and GIP (glucose-dependent insulinotropic polypeptide) are the two major and most important mediators that are secreted in response to food ingestion. GLP-1 acts by binding GLP-1 receptors expressed in the pancreas, heart, blood vessels, gastrointestinal tract, kidneys, lungs, breasts, and central nervous system. GLP-1 receptor agonists suppress glucagon secretion from beta pancreatic cells, decrease gastrointestinal motility, and increase satiety through their action in nervous system. Consequently, the parameters of glycemic control and other metabolic parameters improve [65,66].

GLP-1 receptors were found on cardiac and vascular human tissues, human coronary endothelial cells, and human umbilical vein endothelial cells [65,67]. Liraglutide was shown to increase eNOS and NO production, which are mediated by AMPK-dependent pathways. GLP-1 protects cardiac micro vessels against oxidative stress and apoptosis [65]. Exenatide improved endothelial dysfunction, markers of inflammation, oxidative stress and vascular activation in patients with obesity and pre-diabetes [65]. After infusion of the GLP-1 receptor agonist an improvement of microvascular blood volume and microvascular blood flow was observed in rats, the effect was associated with an increase in circulating NO levels [68]. GLP-1 receptor agonists improved endothelial function in patients with type 2 diabetes mellitus [69,70,71]. Stimulation of endothelial GLP-1 receptors lead to improvement of endothelial function [66]. The infusion of GLP-1 increased forearm blood flow response to acetylcholine by 30% without significant changes of plasma glucose or insulin concentrations [66].

Treatment with GLP-1 receptor agonists decreased systolic and diastolic blood pressure values in patients with diabetes, i.e., systolic blood pressure up to 4.6 mmHg with the liraglutide treatment [67]. Liraglutide thus reduced pulse wave velocity in patients with diabetes [45]. Exenatide was also shown to improve arterial stiffness in type 2 diabetes patients [72,73].

Exenatide was shown to act through sirtuin 1 in ameliorating hepatic steatosis [74]. Stimulation of GLP-1 receptors leads to cAMP generation and activation of downstream pathways (PKA, AMPK and PI3K/AKT; mTOR); the AKT pathway being a major regulator of physiological responses to normal ageing. Activation of these pathways promotes cellular survival [75].

The majority of GLP-1 receptor agonists were shown to act in a cardioprotective manner and to decrease the incidence of major adverse cardiovascular events [32]. The first major trial of cardiovascular outcomes in GLP-1 receptor agonists was ELIXA (Lixisenatide in Patients with Type 2 Diabetes and Acute Coronary Syndrome), where lixisenatide was noninferior compared to placebo in the effect on 3-point MACE (composed of cardiovascular death, nonfatal myocardial infarction and nonfatal stroke) [76]. Liraglutide in the LEADER study (Liraglutide and Cardiovascular Outcomes in Type 2 Diabetes) decreased the incidence of 3-point MACE by 13% compared to placebo [77]. Semaglutide showed similar protective effects on 3-point MACE, which was decreased by 26% compared to placebo in the SUSTAIN study (Semaglutide and Cardiovascular Outcomes in Patients with Type 2 Diabetes) [78]. Dulaglutide decreased the incidence of 3-point MACE by 12% in the REWIND study, being the one that included mostly patients with diabetes with cardiovascular risk factors and not overt cardiovascular diseases [79].

#### 5.1.4. Acarbose

Acarbose lowers postprandial glucose excursions through the inhibition of alpha glucosidase, located in the brush border of the small intestine, thus delaying the absorption of dietary carbohydrates. Nowadays, it is rarely prescribed due to its adverse gastrointestinal effects and mild effects on glycemic control [80]. Acarbose prevented the impairment of postprandial endothelial function in type 2 diabetes patients [81,82]. In another study, acarbose was not proven to influence endothelial function in type 2 diabetes patients [83]. Acarbose was shown to improve arterial stiffness in type 2 diabetes patients through its action on vascular remodeling parameters [84]. It also increased lifespan in mouse models [85]. Similar studies have not yet been performed in humans. Acarbose did not influence the risk of major adverse cardiovascular events in Chinese patients with coronary heart disease and impaired glucose tolerance [86].

### 5.2. Other Drugs

#### 5.2.1. Acetylsalicylic Acid (Aspirin)

Acetylsalicylic acid acts through cyclooxygenase-1 (COX-1) acetylation, which results in the reduced production of thromboxane A2. This leads to reduced platelet reactivity, thus contributing to the prevention of thrombosis and atherosclerosis. Nevertheless, acetylsalicylic acid has an anti-inflammatory action that is mediated thorough the inhibition of COX and indirectly through modulation of NFκB, which has been shown to be the main molecule to orchestrate ageing and diabetes associated arterial wall damage [87]. Additionally, it prevents EC senescence by increasing the bioavailability of NO and its antioxidative action [88,89].

Acetylsalicylic acid has been proven to reduce the incidence of further cardiovascular events in patients with diabetes in secondary prevention, while its role in primary prevention is questionable and currently not recommended by the guidelines. Therefore, its role in prevention of arterial ageing per se remains to be further studied.

#### 5.2.2. Statins

Statins are lipid lowering drugs that inhibit cholesterol synthesis through inhibition of HMG-CoA reductase. Their main effect is low density lipoprotein (LDL) reduction. In addition to the well-known effects, statins possess additional beneficial pleiotropic effects that are beyond their primary mode of action. Mechanisms of these beneficial effects are probably mediated through their interference with the synthesis of isoprenoid intermediates [90]. Through these, they reduce oxidative stress, upregulate the eNOS/NO pathway and reduce inflammatory pathways [91]. In addition, they reduce the production of advanced glycation end products (AGEs), particularly in patients with diabetes [92]. They also upregulate the Nrf2 pathway, thus inducing antioxidative pathways as well as reducing AGE associated VSMC proliferation [93]. Through all the described actions, they act beneficially on endothelial function, reduce arterial stiffness and consequently reduce major cardiovascular events, especially in the ageing population [89,90,93]. In addition, statins could slightly activate several longevity genes, such as sirtuins, AMPK, Klotho etc. [94].

#### 5.2.3. Renin-Angiotensin-Aldosterone System Inhibitors

Angiotensin convertase enzyme (ACE) inhibitors and angiotensin receptor blockers (ARBs) have their primary role in blood pressure reduction. Nevertheless, the two main macrovascular ageing components, i.e., endothelial dysfunction and arterial stiffness, are also associated with RAAS hyperreactivity. Therefore, it is expected, that treatment with these two drugs could influence arterial aging per se [95]. The effects of ACE inhibitors on arterial ageing is at least in part independent of blood pressure reduction. ACE inhibitors through increase in bradykinin in the arterial wall, reduce oxidative stress and increase NO bioavailability. Additionally, both ACE inhibitors as well as ARBs reduce collagen deposition, thus increasing the ratio between elastin and collagen in the arterial wall. This leads to better arterial compliance and delayed arterial ageing [90,96]. There is also scarce data that aldosterone antagonists (spironolactone and eplerenone) reduce arterial stiffness through the increase of elastin-collagen ratio [90].

#### 5.2.4. New Emerging Therapies

Rapamycin acts as an inhibitor of the mTOR pathway that is characterized by increased EC senescence and endothelial dysfunction [97]. Its inhibition leads to improvement in endothelial function through increase in NO bioavailability and reduced oxidative stress [98]. In animals, it has been shown to also reduce mitochondrial oxidative stress and increase the expression of various endogenous antioxidant enzymes [99]. Additionally, it diminishes the inflammatory processes in the arterial wall, particularly through downregulation of NFκB-mediated processes [100]. Rapamycin has been successfully used as an immunosuppressant, while its preventive role in ageing of the cardiovascular system with or without diabetes is emerging [101].

Resveratrol is an antioxidative substance that has been abundantly studied in association with ageing. Its use has been shown to improve endothelial dysfunction through increase in NO bioavailability and reduction in oxidative stress parameters [102]. Particularly, it has a positive effect on upregulation of the superoxide dismutase, an anti-oxidative enzyme, as well as the oxidative stress related defense mechanism mediated through Nrf2 genes. Additionally, it has been shown to inhibit the NFκB pathway, thus acting as an anti-inflammatory [103,104]. Nevertheless, its regular use still remains to be seen. Resveratrol is also a potent activator of sirtuin 1 [105]. In line with this resveratrol action, small molecules—sirtuin activators are even more potent and therefore very promising agents in this regard [106].

AGEs could also be nonenzymatically broken by the use of the AGE crosslink blockers (alagebrium chloride) or their formation prevented by the use of aminoguanidine. Both therapies are still in the research process. If ever fully used in clinical practice, they could both be used to reduce arterial stiffness. Additionally, drugs acting on the blockage of AGE receptors or as sham receptors are also under development [107]. These therapies seem promising, as diabetes and hypertension accelerate the formation of AGEs by nonenzymatic glycation of proteins, particularly collagen, which then crosslink and increase arterial stiffness [90,108]. Thus, AGEs could potentially influence the arterial ageing process as well.

Our study group proposed and studied a new, innovative approach in ameliorating or preventing arterial wall deterioration caused by arterial ageing in diabetes [109]. We proposed a short-term 1-month treatment with a low-dose combination of statin (fluvastatin, 10 mg daily) and sartan (valsartan, 20 mg daily), which improved endothelial function and reduced arterial stiffness in type 1 and type 2 diabetes patients [110,111]. We have also shown that the effects of this treatment persisted for several months after its discontinuation. The beneficial effects of the treatment approach on endothelial function and arterial stiffness improvement were achieved again after treatment repetition, allowing for the so-called cyclical approach [112]. It is based on a huge amount of data proving that the drugs used induce significant pleiotropic effects on the arterial wall [113]. Additionally, we found that a low-dose combination of fluvastatin and valsartan acted through the expression of age-associated genes and telomerase activation that are significantly associated with improvement of endothelial dysfunction and arterial stiffness, thereby revealing the significant anti-ageing capability of low-dose combination [114,115].

## 6. Possible Clinical Implications

**Figure 3:**
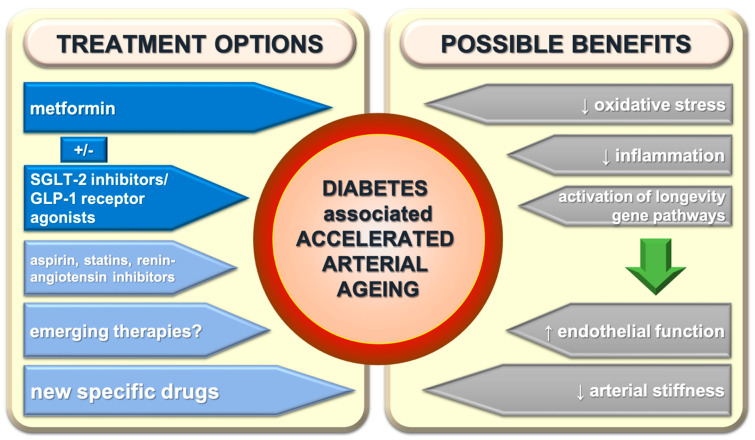
Treatment possibilities of diabetes-associated accelerated arterial ageing and its effects. SGLT-2—sodium-glucose cotransporter-2; GLP-1—glucagon like peptide-1.

Current knowledge regarding the beneficial effects of available drugs on arterial ageing in patients with diabetes is still insufficient. However, based on current knowledge, it could be speculated that metformin has powerful effects and, if so, metformin should be an obvious part of treatment of patients in type 2 diabetes. It is very likely that SGLT-2 inhibitors and GLP-1 receptor agonists also have important effects, but it is also very likely that their effects on arterial ageing differ significantly; further studies are needed. If this assumption turns out to be correct, a more efficient agent could have an advantage over another one. Other antidiabetic drugs seem to have much lower anti-ageing effects on the arterial wall, if any at all. Taking these assumptions into account, the combination of metformin and SGLT-2 inhibitor or GLP-1 receptor agonist (the more beneficial agent remains to be revealed in future) would be the most appropriate treatment among antidiabetic drugs, regarding arterial ageing in patients with diabetes. Whether there are any additive or even synergistic effects between metformin and SGLT-2 inhibitors or GLP-1 receptor inhibitors remains to be explored. The effects of aspirin, statins, and inhibitors of the renin-angiotensin system on arterial ageing are probably slight or mild and therefore could not additionally guide their use in patients with diabetes. In the absence of specific treatment, several described emerging treatments should be intensively studied (Figure 3). Anyhow, it seems plausible to speculate that focusing on arterial ageing is an appropriate new approach in diabetes, and that in the future, a new specific treatment targeting arterial ageing in patients with diabetes would be available and will decrease the rate of diabetic complications.

## 7. Conclusions

In diabetes, the usual process of arterial ageing is accelerated. Both, diabetes and arterial ageing induce pathological effects on the arterial wall, although by different basic mechanisms. Collectively, this leads to aggravation of arterial wall injury and dysfunction, and consequently accelerated cardiovascular events in patients with diabetes. Therefore, assessment and prevention of arterial ageing per se, in addition to diabetes management, appears to be valuable for preventing cardiovascular complications. Several antidiabetic drugs were shown to have protective effects on implicated aspects of arterial ageing in diabetes, particularly metformin, SGLT-2 inhibitors, and GLP-1 receptor agonists. However, their exact roles, mechanisms, and effectiveness in arterial ageing remain to be extensively studied in the near future. Nevertheless, enough evidence exists for metformin to have secured its place as the first line therapy in type 2 diabetes. It should probably be followed by SGLT-2 inhibitors or GLP-1 receptor agonists. Furthermore, these drugs should be prescribed as early as possible in the course of diabetes, as their greatest beneficial potential on arterial aging should probably be expected early after diagnosis. Other drugs seem promising as well. Thus, it seems that arterial ageing should be considered in all patients with diabetes and effective treatment targeting both diseases should be sought in order to ameliorate diabetes complications in generally longer living populations.

## Data Availability

Not applicable.
